# Septic Pylephlebitis in the Setting of COVID-19 Infection: A Case Report

**DOI:** 10.7759/cureus.53240

**Published:** 2024-01-30

**Authors:** Anosh Khan, Montaser Alrjoob, Mahrukh A Khan, Peter Fish, Amer Abu-Shanab

**Affiliations:** 1 Department of Internal Medicine, Monmouth Medical Center, Long Branch, New Jersey, USA

**Keywords:** thrombosis, hypercoagulability, covid-related hypercoagulability, liver abscess, pylephlebitis, covid-19

## Abstract

Portal vein thrombophlebitis is a rare complication that can occur in various hypercoagulable states, including COVID-19. We are presenting a 74-year-old female with a history of hypertension, diabetes, and lymphoma who contracted the COVID-19 infection and presented with persistent fever, leukocytosis, and mild epigastric tenderness. She developed hypotension, acute hypoxic respiratory failure, and worsening leukocytosis with bandemia and was diagnosed with portal vein thrombosis (PVT) and superior mesenteric vein thrombosis. The patient received broad-spectrum IV antibiotics and full anticoagulation therapy with heparin and was discharged on oral Warfarin after completing 14-day antibiotic therapy. She presented again with recurrent watery diarrhea, fever, abdominal pain, and fatigue and was diagnosed with pylephlebitis and multiple small liver abscesses. The patient was treated with antibiotics for six weeks and was discharged on warfarin, furosemide, and spironolactone with close outpatient follow-up. Prolonged fever in COVID-19 patients can indicate extensive thrombosis at unusual sites, which can lead to major morbidity and mortality in patients.

## Introduction

Portal vein thrombophlebitis, a narrowing or blockage of the portal vein by a blood clot, is a complication of intra-abdominal or pelvic infection that usually starts with thrombophlebitis of the small veins that drain the site of infection and extends to larger veins, leading to thrombophlebitis of the portal vein [1]. A combination of thrombophlebitis of the portal venous system (PVS) and superimposed bacterial infection can develop, labeled septic pylephlebitis, which is usually seen as a complication of diverticulitis and appendicitis [2]. Additional risk factors include hypercoagulable states and clotting factor deficiencies. With a mortality rate reaching 25%, early detection and management are vital to prevent severe complications such as liver abscess or venous ischemia [2]. A rare case of recurrent *Streptococcus mitis* bacteremia secondary to septic pylephlebitis in the context of COVID-19 and *Clostridium difficile* infections is reported here.

## Case presentation

Our patient is a 74-year-old female with a past medical history of osteoarthritis, diabetes mellitus type 2, and hypertension who presented to the emergency department for evaluation of fever, fatigue, loss of appetite, and watery diarrhea, which were progressively worsening for the last 12 days. The patient denied any travel history, sick contacts, changes in diet, or recent hospitalizations, but she had a dental extraction one week before presentation. In addition, the patient had tested positive for SARS-CoV2 via PCR 10 days before presentation. 

On presentation, the patient was hypotensive with a blood pressure of 89/56, a heart rate of 71, a respiratory rate of 22, a temperature of 100.9, and saturating above 90% on room air. A physical exam was significant for decreased breath sounds in bilateral lung bases and acute tenderness upon deep palpation of the epigastrium. Initial laboratory studies were significant for a WBC count of 14.4. Electrolytes were all within normal limits. Liver function tests (LFTs) showed elevated ALT, AST, and ALP. C-reactive protein, pro-calcitonin, and lactic acid were also elevated. Urinalysis was unremarkable. The hepatitis panel was negative. A summary of the patient's blood work can be found in Table 1. 

**Table 1:** A summary of patient's CMP and CBC at first and second admission CMP: Comprehensive metabolic panel, CBC: Complete blood count, HCT: Hematocrit, HGB: Hemoglobin, RBC: Red blood cell, WBC: White blood cell, CRP: C-reactive protein, AST: Aspartate transaminase, ALT: Alanine transaminase, GFR: Glomerular filtration rate, BUN: Blood urea nitrogen

CMP	Latest Reference Range & Units	First admission	Second admission
Sodium	135.0 to 145.0 mEq/L	144.0	143.0
Potassium	3.5 to 5.2 mEq/L	4.6	3.1 (L)
Chloride	99.0 to 109.0 mEq/L	113.0 (H)	107.0
CO2	24 to 35 mEq/L	14 (L)	20 (L)
Anion Gap	8 to 16 mEq/L	17 (H)	16
BUN	5 to 21 mg/dL	21	20
Creatinine	0.40 to 1.10 mg/dL	1.07	1.33 (H)
GFR	>=60 mL/min/1.73m2	>60	42 (L)
ALT	10 to 43 Unit/L	294 (H)	39
Albumin	3.5 to 5.0 g/dL	3.5	3.4 (L)
Alkaline Phosphatase	42 to 119 Unit/L	185 (H)	353 (H)
AST	13 to 41 Unit/L	344 (H)	45 (H)
Bilirubin, Total	0.2 to 1.2 mg/dL		1.4 (H)
Bilirubin, Direct	0.0 to 0.3 mg/dL	0.9	0.7 (H)
Calcium	8.3 to 10.2 mg/dL	7.6 (L)	8.4
Glucose	70 to 110 mg/dL	207 (H)	168 (H)
Magnesium	1.5 to 2.5 mg/dL	2.0	0.7 !!
LACTIC ACID	0.5 to 2.0 mmol/L	9.4 !!	3.8 (H)
CRP, High Sensitivity	<=7.00 mg/L	209.63 (H)	166.21 (H)
Procalcitonin	<=0.50 ng/mL	130.42 !!	
CBC	Latest Reference Range & Units	First admission	Second admission
WBC	4.5 to 11.0 K/CMM	14.4 (H)	18.5 (H)
RBC	4.00 to 5.30 M/CUMM	3.45 (L)	3.52 (L)
HGB	12.0 to 15.5 g/dL	10.3 (L)	10.0 (L)
HCT	38.0 to 46.0 %	31.6 (L)	30.9 (L)

**Figure 1:**
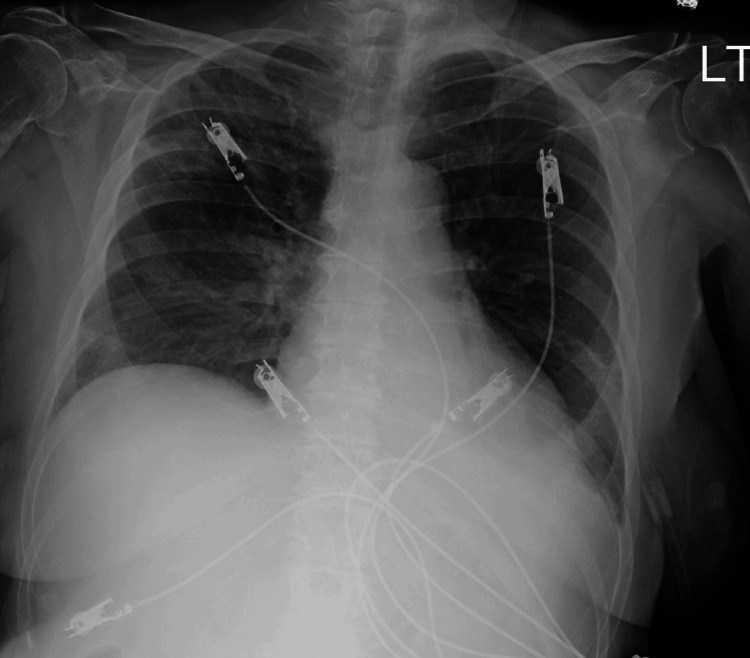
CXR at presentation shows stable lungs with bilateral interstitial pulmonary infiltrates without demarcated consolidations

**Figure 2:**
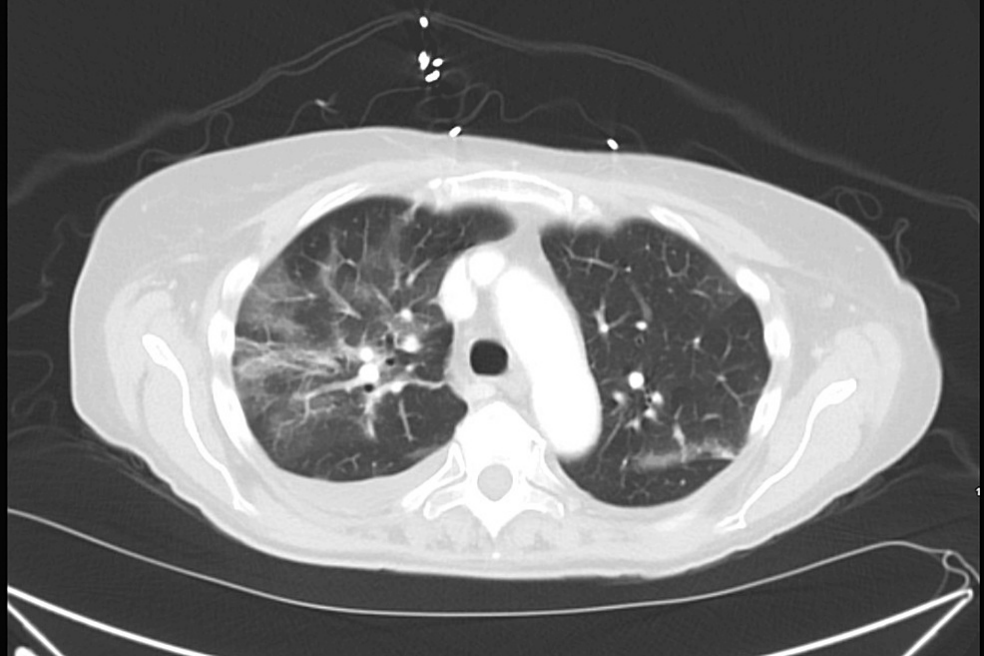
Lung CT scan showing ground-glass opacities throughout the lungs

The chest X-ray showed bilateral interstitial pulmonary infiltrates concerning COVID-19 pneumonia (Figure 1). Blood cultures were collected, and the patient was treated with intravenous vancomycin and piperacillin-tazobactam. A CT scan of the chest, abdomen, and pelvis with contrast demonstrated peripheral ground glass opacities throughout bilateral lungs as well as portal venous thrombosis with portal gas extending within superior mesenteric venous tributaries (Figure 2). The patient was also noted to have a moderate amount of abdominopelvic ascites with evidence of pneumatosis and wall thickening in some segments of small bowel loops concerning ischemic enteritis and/or pseudomembranous colitis. Two sets of peripheral blood cultures on admission came back positive for *Streptococcus mitis*. 

The patient was treated with a 14-day course of Piperacillin-Tazobactam as well as a Heparin infusion for anticoagulation of portal vein thrombosis. Ultrasound abdomens showed the presence of portal vein thrombosis with moderate ascites. Paracentesis was performed, and fluid analysis showed a normal WBC count and was found to be negative for malignant cells. Ascites fluid culture was negative as well. An esophagogastroduodenoscopy (EGD) with tissue biopsy of the antrum was negative for *H. pylori* or intestinal neoplastic changes. Transthoracic echocardiography (TTE) and subsequent transesophageal echocardiography (TEE) were also unremarkable; thus, infective endocarditis was excluded. Although the extensive diagnostic workup was inconclusive, the patient was discharged on oral warfarin for anticoagulation after completing a 14-day antibiotic course. Repeat blood cultures were negative at the time of discharge. 

Shortly after discharge, the patient presented to the emergency department again with recurrent watery diarrhea, fever, abdominal pain, and fatigue. On arrival, the patient was noted to be hypotensive with a blood pressure of 65/40, a heart rate of 100, and a respiratory rate of 16, saturating around 95% on a 5 L nasal cannula. Blood work was significant for an elevated WBC of 18.5. Serum potassium and magnesium were decreased, while creatinine and lactic acid were increased. LFTs showed ALT and AST within normal limits, but ALP was elevated (Table 1). Examination findings were pertinent only for tenderness to palpation of the upper abdominal quadrants. Blood cultures were positive for *Streptococcus mitis*. The patient also tested positive for *Clostridium difficile* (*C. diff*). Intravenous ceftriaxone and oral vancomycin were started, and warfarin was given a high INR. The patient was started on a heparin infusion with close monitoring of the PT/INR. Repeat chest X-rays showed bilateral linear opacities like prior, in addition to mild-moderate bilateral pleural effusions. Ultrasound abdomens showed persistent portal vein thrombosis. A thrombophilia profile workup including Factor V Leiden, Protein C, and S deficiency was negative. A repeat CT scan with contrast of chest, abdomen, and pelvis demonstrated thrombus and gas in the portal vein with extension in the superior mesenteric vein (SMV); mesenteric edema and abdominopelvic ascites were also increased compared to the prior exam. Ground-glass opacities with new bilateral pleural effusions were also present. Diagnostic and therapeutic paracentesis was done. Ascitic fluid was transudative in studies and remained negative for infection or malignancy.

**Figure 3:**
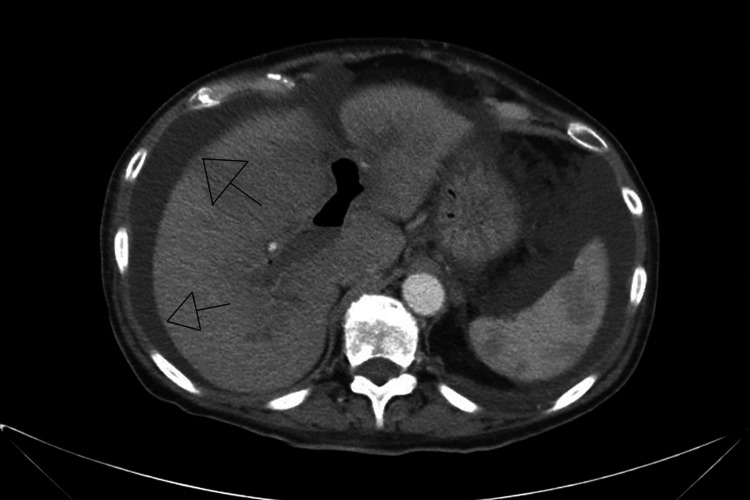
Liver abscess seen in an abdominal CT scan

Due to the high suspicion of a gastrointestinal source of infection, a scintigraphy study of the liver and spleen was done, which was positive for pylephlebitis and multiple small liver abscesses (Figure 3). Meanwhile, repeat peripheral blood cultures showed no growth, and the patient had clinical improvement. The patient was ultimately discharged on warfarin, furosemide, and spironolactone, with an antibiotic course of amoxicillin-clavulanic acid for a total of six weeks with oral vancomycin to prevent further worsening of the *C. diff* infection. The plan was to follow up outpatient in the next 4-6 weeks to evaluate for resolution of PVT and pylephlebitis.

## Discussion

Pylephlebitis is a rare complication of intra-abdominal infections that causes a suppurative infection of the porto-mesenteric venous thrombus with a mortality rate greater than 20% [1]. It is associated with appendicitis, diverticulitis, IBD, cholecystitis, pancreatitis, or recent abdominal surgeries. Symptoms can be variable and nonspecific, including fever, abdominal pain, vomiting, diarrhea, anorexia, hepatomegaly, splenomegaly, and ascites. Leukocytosis and elevated inflammatory markers are observed in over 80% of cases. Elevated liver enzymes, hyperbilirubinemia, and anemia can also be seen in some patients [1,3-5].

The initial trigger of the coagulation cascade in pulmonary vessels caused by a local cytokine storm and endothelial layer dysfunction can cause a hypercoagulable effect in the micro and macrovasculature due to the magnification of the immune response under COVID-19 infection. Portal and mesenteric vein thrombus (PVT and MVT) is a rare but reported complication during acute or post-COVID infection ranging from the 1st week to one month [5,6]. An intra-abdominal infection with potential extension into the portal venous system in a concurrent hypercoagulable state provides a nidus for enteric commensals to form septic thrombophlebitis. Positive blood culture is often seen in more than half of the cases, with the most common single isolates being *Bacteroid* species, *E. coli*, and *Streptococcus* species [1,3]. In our case, the recurrence of *Streptococcus mitis* bacteremia is likely due to the presence of PVT from COVID-19, which acted as a nidus for infection.

A timely and appropriate diagnosis is important, yet it is usually delayed due to a series of factors. Abdomen ultrasound can often be limited due to the presence of normal bowel gas and operator reporting. CT abdomen with contrast is the second modality of choice. Phylephlebitis is often difficult to visualize compared to PVT. Findings indicative of septic thrombophlebitis include portal vein dilatation proximal to occlusion with wall thickening, portal venous gas, the presence of collateral vessels, and complications of hepatic abscess or intestinal ischemia [7,8]. FDG-PET and hepatic Tc-99 scintigraphy can be used as adjuncts for diagnostic confirmation in limited cases [1].

Empiric treatment with broad-spectrum antibiotics is the initial approach, which is continued for at least six weeks. Parenteral broad-spectrum antibiotics should be continued at least for the first two weeks and then transition to oral antibiotics according to culture sensitivity [9]. Percutaneous aspiration and drainage can also be performed in a few subjective cases, although they have an association with a higher risk of mortality and morbidity [7]. A shorter duration of antibiotics on initial admission led to further worsening of symptoms in our case. Meanwhile, studies on the role of anticoagulation are still limited. Naymagon et al. observed a significantly increased rate of PVT resolution and decreased development of chronic portal hypertensive symptoms in anticoagulated individuals [10]. Subjective use of warfarin, heparin, low molecular weight heparin (LMWH), and Factor Xa inhibitors has been observed in the literature. We managed our patient as an outpatient on Warfarin and an inpatient on Heparin drip due to supratherapeutic INR and a potential invasive procedure. There is no standard duration for anticoagulation use. Generally, these anticoagulants are used for 3 to 6 months [10,2]. Follow-up with repeat imaging to assess PVT resolution is critical to prevent further complications like an extension of the thrombus, portal hypertension, liver abscess, septic shock, mesenteric ischemic, and pulmonary emboli [3,4].

The COVID-19 infection has shown its notorious impact on almost every organ system of the body. The higher incidence of pulmonary emboli seen in COVID-19 patients in the past couple of years calls for increased awareness of thrombosis in other unusual sites like porto-mesenteric venous circulation, leading to septic thrombophlebitis [11]. The general symptoms of COVID-19 infection often overlap with the unspecific presentation of pylephlebitis, delaying medical care. Hence, clinicians should keep pylephlebitis in their differential diagnosis, especially in the setting of COVID-19 infection, to ensure early diagnosis, treatment, and prevention of complications. In our case, we think that COVID-19 contributed to the increased hypercoagulability state in the patient, which played a major role in the portal vein thrombophlebitis formation, and superimposed him for bacterial pneumonia [11].

## Conclusions

Septic pylephlebitis, accompanied by *Streptococcus mitis* bacteremia and *Clostridium difficile*, is an uncommon complication of the COVID-19 infection. If left undetected or inadequately treated, it can lead to the formation of liver abscesses. The general symptoms of COVID-19 often mimic the non-specific presentation of pylephlebitis, potentially causing a delay in receiving proper medical care. Therefore, timely identification and effective management of septic pylephlebitis involving prolonged antibiotic use and anticoagulation are essential to prevent such complications.
